# Empowering Social Growth Through Virtual Reality–Based Intervention for Children With Attention-Deficit/Hyperactivity Disorder: 3-Arm Randomized Controlled Trial

**DOI:** 10.2196/58963

**Published:** 2024-10-28

**Authors:** Ka Po Wong, Bohan Zhang, Cynthia Yuen Yi Lai, Yao Jie Xie, Yan Li, Chen Li, Jing Qin

**Affiliations:** 1 Department of Applied Social Sciences The Hong Kong Polytechnic University Hong Kong China (Hong Kong); 2 Centre for Smart Health School of Nursing The Hong Kong Polytechnic University Hong Kong China (Hong Kong); 3 Department of Rehabilitation Sciences The Hong Kong Polytechnic University Hong Kong China (Hong Kong); 4 School of Nursing The Hong Kong Polytechnic University Hong Kong China (Hong Kong); 5 Department of Computing The Hong Kong Polytechnic University Hong Kong China (Hong Kong)

**Keywords:** attention deficit and hyperactivity disorder, virtual reality, social skills, social skills training, emotional control, social growth, digital world, social learning theory

## Abstract

**Background:**

Attention-deficit/hyperactivity disorder (ADHD) usually begins in childhood and is often accompanied by impairments in social functioning. Virtual reality (VR) has emerged as an adjunctive tool to embed in social skills training to enhance the social skills of children with ADHD, but its effectiveness requires further investigation.

**Objective:**

This study aims to enhance the social skills of children with ADHD by examining the feasibility and effectiveness of VR-based training in comparison to traditional social skills training.

**Methods:**

A 3-arm randomized controlled trial was conducted with 90 children with ADHD aged 6-12 years. Participants were randomly assigned to 3 weeks of 12-session VR-based social skills training, traditional social skills training, or a waitlist control group of equivalent duration. Outcome measures included assessments by a clinical psychologist who was blinded to group assignments, the Social Skills Improvement System Rating Scale, the Behavior Rating Inventory of Executive Function, and the Simulator Sickness Questionnaire, conducted at baseline and after the intervention.

**Results:**

The preliminary results support the feasibility and acceptability of VR training for children with ADHD aged 6-12 years. Analysis showed that the VR and traditional social skills training groups experienced a statistically significant improvement in the clinical psychologist assessment of social skills and parent-rated self-control, initiative, and emotional control after the intervention compared with baseline. The VR group performed significantly better than the traditional social skills group on social skills assessed by clinical psychologists (*F*_2,85_=76.77; *P*<.001) and on parent-rated self-control (*F*_2,85_=18.77; *P*<.001), initiative (*F*_2,85_=11.93; *P*<.001), and emotional control (*F*_2,85_=17.27; *P*<.001). No significant between-group differences were found for parent-rated cooperation and inhibition (all *P*>.05).

**Conclusions:**

The findings provide preliminary evidence supporting the feasibility and superior effectiveness of VR-based social skills training compared to traditional approaches for enhancing social skills and related executive functions in children with ADHD. These results suggest that VR may be a valuable tool to embed within social skills interventions for this population. Further research is warranted to explore the long-term impacts and generalizability of these benefits.

**Trial Registration:**

ClinicalTrials.gov NCT05778526; https://clinicaltrials.gov/study/NCT05778526

**International Registered Report Identifier (IRRID):**

RR2-10.2196/48208

## Introduction

### Overview

Attention-deficit/hyperactivity disorder (ADHD) is one of the children’s most common neurobehavioral disorders [1], with a global prevalence of 5.3% [2] and a prevalence of 7.6% among children aged 3-12 years [3]. Inattention, impulsivity, and hyperactivity are the 3 main categories of ADHD symptoms, which are associated with nontypical cognitive and behavioral functioning due to their influence on the regulation of emotions and brain networks. Persistent patterns of inattention, impulsivity, and hyperactivity that interfere with daily functioning are considered ADHD [4]. Meanwhile, these symptoms may contribute to social difficulties and peer problems due to a deficit in social executive function skills [5].

Children with ADHD often experience specific social skills difficulties that can affect their interactions and relationships with others [6]. These children may struggle to think before acting, leading to impulsive behaviors that can disrupt conversations or be seen as socially inappropriate [7]. They may have difficulty considering the consequences of their actions, leading to difficulty in maintaining self-control during social interactions. They also have trouble sustaining attention during conversations and missing important details or cues [8]. They often move excessively, are restless, or have difficulty staying in a particular place, making it difficult to actively participate in group activities, take turns, or engage in cooperative play [9]. Furthermore, difficulty regulating emotions can affect social interactions in children with ADHD [10], leading to outbursts of anger, frustration, or impatience. These emotional challenges can strain relationships with peers and make it difficult to respond effectively to social situations. This persistent social impairment can lead to peer rejection and exacerbate academic failure, depression, and anxiety [11,12]; thus, addressing social impairment during childhood is critical.

Social skills training is one of the ubiquitous approaches to ameliorating social behaviors in people with ADHD. The training usually involves a combination of didactic instruction and role playing activities [13]. Effective social skills interventions offer strategies and support to help children with ADHD improve their social competence, navigate social situations better, and foster positive relationships [14]. However, this method may be restricted by time and space, and imagining actual situations during training is difficult for children with ADHD.

Virtual reality (VR)–based interventions are considered an effective alternative to traditional social skills training, providing the immersion of a digital environment and engaging users in the learning process [15]. VR can produce various scenarios that are not easily and safely operated in contrast to equivalent real-life training programs [16,17]. Typical VR-based interventions for ADHD use immersive digital environments that simulate real-world social and academic situations. For example, these interventions may place the child in a classroom setting with various auditory (eg, classmates chatting and teacher lecturing) and visual (eg, posters on walls and other students moving around) distractions [18,19]. By requiring the child to maintain focus and inhibit responses to these distractions within the VR environment, the intervention aims to improve their inhibitory control and attention regulation skills [20]. The VR setting allows the child to practice these skills in a safe, controlled space without the consequences of real-world mistakes.

Additionally, VR interventions may present the child with digital social scenarios, such as interacting with digital peers or authority figures. These scenarios allow the child to observe and practice appropriate social behaviors, like taking turns, making eye contact, and responding to social cues, which can be challenging for children with ADHD [21]. Immediate feedback and the ability to repeat scenarios within VR can help consolidate these social skills.

By incorporating these types of immersive VR environments, the interventions leverage the unique affordances of VR to target the core cognitive, behavioral, and social deficits associated with ADHD engagingly and safely. Social skills training using VR technology exists, and the efficiency of such applications has been demonstrated; however, beneficiaries are mainly those with autism spectrum disorders, schizophrenia, and intellectual disabilities [22-25]. A VR-based intervention to enhance the social interaction skills of children with ADHD is limited, and its effectiveness has not been empirically studied.

### Theoretical Background

The VR-based social skills intervention is rooted in social learning theory, as developed by Bandura [26]. Social learning theory posits that individuals can acquire new behaviors, attitudes, and emotional responses by observing the actions and consequences experienced by others [27]. This observational learning process is a key mechanism by which children develop social competence and navigate complex interpersonal situations. For children with ADHD, deficits in social skills are often linked to challenges with attention, impulse control, and emotional regulation—factors that can hinder their ability to effectively observe, process, and learn from social cues and interactions [28]. The immersive nature of VR provides an ideal platform to address these challenges and leverage the principles of social learning theory [15].

Within the VR environment, children with ADHD can observe modeled social behaviors and their consequences in a safe, controlled setting. This allows them to focus on the relevant social cues and responses without the distractions or anxiety that may arise in real-world interactions [29]. The VR-based intervention can facilitate observational learning and cognitive processing of adaptive social behaviors by presenting diverse social scenarios and modeling appropriate social skills.

The VR-based intervention incorporates opportunities for active practice and role playing of these observed social skills. This aligns with Bandura's emphasis on the importance of engaging in enactive learning, where individuals have the chance to rehearse and refine new behaviors [26]. The immediate feedback and reinforcement provided within the VR environment can further support the acquisition and consolidation of social skills. Importantly, social learning theory also highlights the role of self-efficacy—an individual’s belief in their ability to successfully execute a behavior [29]. By providing a supportive, scaffolded learning environment in VR, the intervention aims to enhance children’s confidence and self-efficacy in navigating social situations, ultimately promoting the generalization of social skills to real-world contexts.

By incorporating social learning theory [26] into VR-based social skills training, an effective and engaging learning environment that aligns with the unique needs and challenges of children with ADHD can be provided. The VR-based approach leverages the power of observation, modeling, reinforcement, and cognitive processes to promote the acquisition and generalization of adaptive social skills, empowering children with ADHD to navigate social interactions with greater confidence and success.

### Rationales of VR-Based Social Skills Training for ADHD

While traditional social skills training has been used to help children with ADHD, they may be limited by logistical constraints and difficulty simulating real-world social scenarios [13,14]. VR technology offers a promising alternative, as it can immerse participants in customizable social environments that are difficult to replicate in physical settings [15-17]. VR-based interventions have been shown to effectively target social skills deficits in populations such as autism spectrum disorder and schizophrenia [22-25].

However, research on the use of VR-based social skills training for children with ADHD is limited. A few existing studies have focused on using VR to address ADHD symptoms like inattention and impulsivity but have not specifically examined the impact on social skills [30]. Given the unique social challenges faced by this population and the potential benefits of VR-based training, there is a clear need for rigorous investigation into the feasibility and efficacy of VR-based social skills interventions for children with ADHD.

This study aims to address this gap by examining the feasibility and effectiveness of VR-based social skills training for children with ADHD, in comparison to a traditional social skills training approach and a waitlist control group. Drawing on social learning theory, the VR-based intervention is designed to leverage the immersive, engaging features of VR to enhance observational learning, skill practice, and self-efficacy in navigating social situations. Findings from this randomized controlled trial (RCT) will provide valuable insights into the potential of VR-based interventions to improve social competence in children with ADHD.

## Methods

### Study Design

In this study conducted at The Hong Kong Polytechnic University (ClinicalTrials.gov NCT05778526), a 3-arm RCT was implemented from November 2023 to February 2024. The participants were assigned randomly to 3 groups: the VR training group, the traditional social skills training group, and the waitlist control group, maintaining a 1:1:1 ratio. The VR training group received 12 sessions for 3 weeks, with each session lasting 20 minutes. Similarly, the traditional social skills training group also underwent 12 sessions in 3 weeks, with each session lasting 20 minutes. These intervention durations and frequencies align with the findings of a study by Willis et al [14], which reported intervention durations ranging from 2 to 12 weeks and weekly time commitments of 60-90 minutes. Therefore, the chosen intervention duration and frequency in our study are considered appropriate based on existing research. Participants randomized to the waitlist control group were offered VR training after 1 month when data were collected. The guardians of the participants were reminded to attend the training via phone in the first 4 sessions and then via WhatsApp (Meta Platforms) or WeChat (Tencent Holdings Ltd) in the fifth and eighth sessions, respectively. There were no follow-up reminders issued in the following 4 sessions. The protocol of the RCT was published [31]. The study followed the CONSORT (Consolidated Standards of Reporting Trials) guidelines (Multimedia Appendix 1).

### Ethical Considerations

This study received approval from the institutional review board at The Hong Kong Polytechnic University (HSEARS20221221003) and was conducted from November 2023 to February 2024. It adhered to the principles outlined in the Declaration of Helsinki, which included ensuring participant anonymity and obtaining informed consent from the guardians of the participants. Participation was voluntary. Informed consent was obtained through a clear process, ensuring that guardians fully understood the study’s purpose, procedures, and potential risks. Participants were also given the option to withdraw from the study at any time without penalty. Data collected during the study were anonymized to protect participant confidentiality.

### Recruitment

Participants were recruited from the children and youth community centers through extensive advertising, including posters and advertisements on social media. A web-based registration form was created for the interested guardian to register. Interested guardians who provided written informed consent were further evaluated for inclusion and exclusion criteria.

Eligibility criteria for participants were as follows: (1) aged between 6 and 12 years; (2) Chinese ethnicity; (3) residing in Hong Kong; (4) having received a diagnosis of ADHD by Child Assessment Service in Hong Kong or via private practice; (5) stable on pharmacological and psychological treatment for ADHD 8 weeks before baseline (determined by health care professionals based on medication data and behavioral observation); (6) no initiation or change of pharmacological treatment for ADHD during the intervention period; (7) ability to read Chinese and speak and listen to Cantonese by the child and by at least one of their legal guardian; and (8) willing to provide informed consent by the participants’ legal guardians.

Exclusion criteria were as follows: (1) comorbid autism; (2) mental health disorders; (3) an estimated IQ lower than 85 (using the Wechsler Intelligence Scale for Children—Fourth Edition); (4) autism spectrum disorder (previously diagnosed by health care professionals), (5) comorbid acute psychiatric disorder (previously diagnosed by health care professionals); and (6) severe physical disability (eg, blindness, deafness) or learning disability (eg, dyslexia).

### Procedure and Interventions

Potential participants’ guardians completed a telephone prescreening. Following consent, the guardian showed the diagnosis of ADHD of the participants and completed the baseline assessment for the participants. The randomization was conducted using a computer-generator randomizer to generate the random allocation list. The randomization was undertaken by another research assistant not directly involved in the study. A number generated by the computer will be assigned to each eligible subject who will be randomly allocated to the 3 different groups by using the number.

#### VR Training Group

The VR training aimed to enhance the social interaction skills of children with ADHD. Participants in this group wore a head-mounted display and 2 controllers during the training. The intervention included three real-life scenarios, including (1) a classroom and playground, (2) a mass transit railway station and carriages, and (3) a supermarket and restaurant (Multimedia Appendix 2). These scenarios were purposefully designed to simulate real-life social situations, allowing participants to practice and refine their social skills in a controlled and supportive digital environment. Gamification elements were integrated into the immersive VR training, including dynamic simulations and real-time feedback to provide immediate correction of participants’ behaviors.

Each scenario contained approximately 20 or more tasks for the participants to complete. Participants underwent 1 scenario in 1 session. The sequences of the scenarios used in each session were the same for all participants. Each session lasted for a maximum of 20 minutes to ensure the concentration of the participants and prevent any physical effects [32]. The duration was adjusted subject to the emotions of the participants during the training. During the intervention, a research assistant guided the participants in completing the tasks. Multimedia Appendix 3 shows the participants conducting the intervention.

#### Traditional Social Skills Training Group

Participants in this group were educated in social skills through traditional methods, including role-playing activities and didactic instruction. Four modules, including (1) how to introduce yourself and basic social skills, (2) how to listen to others, (3) how to share with others, and (4) learning to know how people feel and how to empathize, were covered in the training, which has been adopted in previous studies [33]. Each participant was taught by 1 instructor. The content of the training and the duration of each session were kept similar to the VR training group.

#### Waitlist Control Group

Participants in this group received no training and were not allowed to change or initiate their medical treatment during the study period.

#### Outcome Measures

Data were collected at 2 time intervals, including baseline (T1) and immediately after the last sessions (T2) to determine the feasibility and effectiveness of the RCT.

#### Primary Outcome: Social Skills

The social skills of the participants were evaluated by the guardians of the participants and a clinical psychologist. The guardians rated using the Social Skills Improvement System-Rating Scales, which has been validated in children with ADHD [34]. A total of 31 items were selected from the self-control, initiative, and cooperation subscales of the Social Skills Improvement System–Parents. The clinical psychologist used a modified version of Riggio basic social skills assessment [35] to evaluate the participants. This involved a 20-minute conversation in which the clinical psychologist observed and assessed their social skills. To minimize unblinding bias during the assessment, each participant will be identified by a case number.

#### Secondary Outcome

##### Executive Functioning

The executive functioning of the participants was measured by the subscale of inhibition (16 items) and emotional control (10 items) of the Behavior Rating Inventory of Executive Function-Parents, which is useful in Chinese children with ADHD [36]. The executive functioning of the participants was evaluated by the guardians of the participants.

##### Motion Sickness

The motion sickness or physical discomfort of subjects using the VR was measured by the Simulator Sickness Questionnaire [37], which was administered at T2 to the participants in the VR training group only.

##### Satisfaction

Satisfaction with training was measured by a 7-point Likert scale administered at T2 to the VR training group.

##### Feasibility and Acceptability Outcomes

Feasibility was assessed through participants’ retention of the intervention. The attendance of the participants during the interventions was recorded, and absence from any training session was considered nonadherence.

##### Justification of Sample Size

A sample size of 20 subjects per group can attain at least 80% power and 90% confidence according to the study of Whitehead et al [38]. We intended to recruit 90 participants (30 per group), assuming a conservative attrition rate of 25% to 30% [19], to reliably determine these outcomes.

### Statistical Analysis

All statistical analyses were performed in the SPSS (version 28, IBM Corp) software and were 2-sided with a level of significance of *P*<.05. The data analysis complied with the principles of intention-to-treat analysis. The subjects’ demographic information was summarized into categorical variables with frequency and percentage. The change between T1 and T2 of each group was tested by a 2-tailed *t* test. Analyses of covariance (adjusted for possible confounding factors) were conducted to evaluate the within-group effects and between-group effects in terms of outcomes. Effect sizes were measured by Cohen *d* and 95% CI. To assess the improvement during the intervention period among the 3 groups, *F* tests were performed on primary and secondary outcome measures at T1 and T2.

## Results

### Participant Characteristics

Between November 2023 and February 2024, we enrolled 108 participants in our study. However, 10 participants were excluded before randomization due to not meeting the inclusion or exclusion criteria, and 8 declined to provide written consent. Consequently, 90 participants, along with their guardians, agreed to participate in the study. These participants and their guardians agreed to join the study and were randomly assigned to the VR training (n=30), traditional social skills training (n=30), or waitlist control group (n=30). Baseline characteristics were balanced among the 3 groups without statistically significant differences (Tables 1 and 2). The mean age for the VR training, traditional social skills training, and waitlist control groups were 8.63 (SD 1.90), 8.30 (SD 1.70), and 8.67 (SD 1.45) years, respectively. All participants attended mainstream schools. Table 1 presents the participants’ baseline demographic characteristics and outcome measure scores, respectively.

**Table 1:** Baseline data for the VRT^a^, TSST^b^, and WLC^c^ groups.

Variables			VRT (n=30)	TSST (n=30)	WLC (n=30)	*P* value^d^	
**Sex, n (%)**							.60
	Male		23 (77)	26 (87)	24 (80)		
	Female		7 (23)	4 (13)	6 (20)		
**Age (years), mean (SD)**			8.63 (1.90)	8.30 (1.70)	8.67 (1.45)	.20	
**ADHD^e^ subtype, n (%)**							.29
	Combined		19 (63)	24 (80)	26 (87)		
	Inattention		7 (23)	4 (13)	3 (10)		
	Hyperactivity or impulsivity		4 (13)	2 (7)	1 (3)		
**Medication, n (%)**							.73
	Yes		18 (60)	17 (57)	15 (50)		
	No		12 (40)	13 (43)	15 (50)		
**Outcomes variables, mean (SD)**							
	**Clinical psychological assessment of social skills**		26.23 (6.40)	25.60 (7.16)	24.23 (6.27)	.49	
	**Social Skills Improvement System–Parents**						
		Self-control	31.57 (4.10)	31.02 (3.79)	31.60 (5.00)	.84	
		Initiative	20.43 (4.91)	19.78 (4.30)	19.65 (4.08)	.77	
		Cooperation	12.07 (1.82)	11.00 (2.68)	11.98 (2.67)	.17	
	**Behavior Rating Inventory of Executive Function–Parents**						
		Inhibition	31.20 (5.46)	32.20 (4.76)	33.05 (6.45)	.44	
		Emotional control	18.53 (4.19)	18.92 (3.35)	19.67 (5.42)	.60	

^a^VRT: virtual reality training.

^b^TSST: traditional social skills training.

^c^WLC: waitlist control.

^d^Chi-square test or *F* test.

^e^ADHD: attention-deficit/hyperactivity disorder.

**Table 2:** Social skills and executive functioning according to the group before and after the intervention

Outcome variables			VRT^a^ (n=30), mean (SD)					TSST^b^ (n=30), mean (SD)					WLC^c^ (n=30), mean (SD)					Within-group analysis, *P* value							Between-group analysis				Effect sizes, Cohen *d* (95% CI)		
			T1		T2		T1			T2		T1			T2		VRT			TSST		WLC		*F* test (*df*)			*P* value	VRT vs TSST			VRT vs WLC
**Clinical psychological assessment of social skills^d^**			26.23 (6.40)		38.20 (2.83)		25.60 (7.16)			30.63 (6.57)		24.23 (6.27)			24.50 (5.17)		<.001			<.001		.73		76.77 (2, 85)			<.001	1.50 (0.91-2.07)			3.29 (2.50-4.06)
**Social Skills Improvement System–Parents^d^**																															
	Self-control	31.57 (4.10)		33.33 (3.56)		31.02 (3.79)			31.93 (2.78)		31.60 (5.00)			31.50 (4.70)		<.001			0.003		.50		18.77 (2, 85)			<.001		0.44 (–0.08 to 0.95)		0.44 (–0.08 to 0.95)	
	Initiative	20.43 (4.91)		21.13 (3.80)		19.78 (4.30)			20.20 (4.02)		19.65 (4.08)			19.60 (3.92)		.01			.001		.59		11.93 (2, 85)			<.001		0.24 (–0.27 to 0.74)		0.40 (–0.11 to 0.91)	
	Cooperation	12.07 (1.82)		12.17 (1.58)		11.00 (2.68)			11.37 (2.16)		11.98 (2.67)			12.03 (2.51)		.33			.05		.61		0.74 (2, 85)			.48		0.42 (–0.09 to 0.93)		0.07 (–0.44 to 0.57)	
**Behavior Rating Inventory of Executive Function–Parents^e^**																															
	Inhibition	31.20 (5.46)		32.00 (4.45)		32.20 (4.76)			33.00 (4.05)		33.05 (6.45)			34.97 (6.75)		.02			.003		<.001		7.10 (2, 85)			.001^f^		–0.24 (–0.74 to 0.27)		–0.52 (–1.03 to –0.00)	
	Emotional control	18.53 (4.19)		16.83 (3.08)		18.92 (3.35)			17.98 (2.68)		19.67 (5.42)			19.63 (4.91)		<.001			<.001		.89		17.27 (2, 85)			<.001		–0.40 (–0.91 to 0.11)		–0.69 (–1.20 to –0.16)	

^a^VRT: virtual reality training.

^b^TSST: traditional social skills training

^c^WLC: waitlist control group.

^d^Higher scores indicate better social skills.

^e^Higher scores indicate poor executive functions.

^f^Heterogeneity variance between independent groups was assessed using Levene test (*P*>.05 indicates no significant difference in variances).

**Figure 1:**
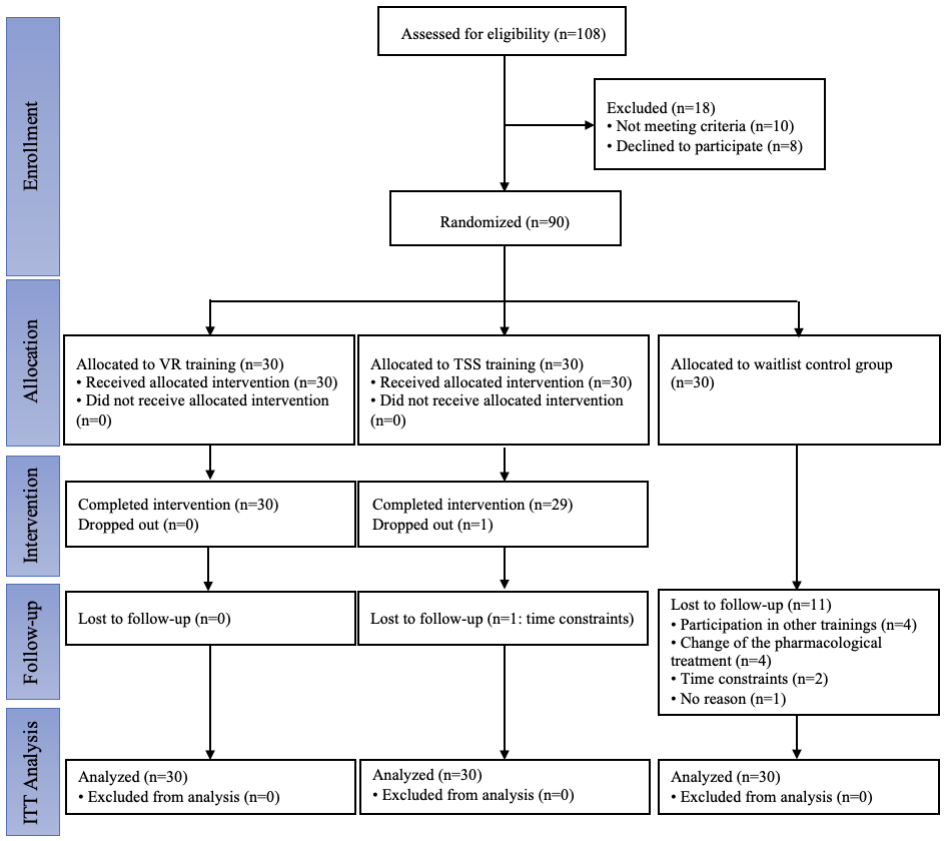
CONSORT (Consolidated Standards of Reporting Trials) flow diagram. ITT: intention-to-treat; TSS: traditional social skills; VR: virtual reality.

During the training, all participants in the VR training group attended 12 sessions, 1 participant in the traditional social skills training group withdrew after 3 sessions due to time constraints, and 11 participants in the waitlist control group withdrew due to participation in other similar trainings, change of the pharmacological treatment, time constraints and loss to follow (Figure 1). Findings provided promising evidence of participants’ acceptance of the VR training.

### Effects of the Interventions on Outcomes

The mean scores for the clinical psychologist assessment of social skills and parent-rated executive functioning and social skills of the 3 groups at T1 and T2 are illustrated in Table 2. Significant improvements between T1 and T2 in the VR training group and the traditional social skills group in terms of clinical psychologist assessment of social skills and parent-rated self-control, initiative, and emotional control were found. Significant deterioration of parent-rated inhibition was also found. No statistically significant difference between T1 and T2 was found in the 3 groups in terms of parent-rated cooperation. In consideration of confounding variables, the assumption of normality, that is, homogeneity of variance between independent groups, was met in clinical psychologist assessments of social skills and parent-rated self-control, initiative, cooperation, and emotional control.

Clinical psychologist assessments of social skills had a statistically significant difference between groups (*F*_2,85_=76.77; *P*<.001), in which the VR training group was 7.57 higher than the traditional social skills training group (*d*=1.50, 95% CI 0.91-2.07) and 13.70 higher than the waitlist control group (*d*=3.29; 95% CI 2.50-4.06).

A statistically significant difference was found between groups (*F*_2,85_=18.77; *P*<.001) regarding parental ratings of self-control, with the VR training group being 1.40 higher than the traditional social skills training group (*d*=0.44; 95% CI –0.08 to 0.95) and 1.83 higher than the waitlist control group (*d*=0.44; 95% CI –0.08 to 0.95). Parent-rated initiative had a statistically significant difference between groups (*F*_2,85_=11.93; *P*<.001), in which the VR training group was 0.93 higher than the traditional social skills training group (*d*=0.24; 95% CI –0.27 to 0.74) and 1.53 higher than the waitlist control group (*d*=0.40; 95% CI –0.11 to 0.91). Parent-rated cooperation had no statistically significant difference between groups.

For parent-rated emotional control, the VR training group was 1.15 lower than the traditional social skills training group (*d*=–0.40; 95% CI –0.91 to 0.11) and 2.80 lower than the waitlist control group (*d*=–0.69; 95% CI –1.20 to 0.16), and this difference was statistically significant (*F*_2,85_=17.27; *P*<.001). The determination of parent-rated inhibition was not feasible due to significant heterogeneity in variance between the independent groups.

Regarding cybersickness in participants in the VR training group, no participant reported discomfort, fatigue, headache, eyestrain, nausea, difficulty concentrating, blurred vision, and dizziness during and after the training. A total of 16 participants were sweating during the VR training. All participants were satisfied with the VR training (mean 6.77, SD 0.43).

## Discussion

### Principal Findings

To our knowledge, this is the first RCT to evaluate the feasibility and acceptability of social skills training for children with ADHD developed through VR technology and clinically assessed by an independent clinical psychologist. Acceptance and satisfaction with the VR training by participants were high. Our findings showed that participants in the VR training and traditional social skills groups experienced significant improvements in social skills, self-control, initiative, and emotional control. Compared with the traditional social skills training group, the VR training group improved more in social skills, self-control, initiative, and emotional control. These results are preliminary; however, the results may be considered useful given their positive impact on social skills and executive function in children with ADHD.

### Comparison With Prior Work

The findings match those observed in earlier studies that VR-based social skills training and traditional social skills training could improve the social skills of children, and VR-based intervention had a better performance than traditional social skills training [25,39]. The results suggest that VR-based interventions may be a promising approach for helping children with neurobiological disabilities, such as ADHD and autism spectrum disorder, acquire social skills due to the immersive and engaging nature of VR. Previous meta-analyses have shown that VR-based interventions are effective in improving cognitive function (ie, learning and attention) in children with ADHD [19,40,41], although these comparisons require caution due to the different purposes of the training and intervention protocols. The focus of our VR training program was to improve children’s social skills, rather than improving their cognitive function as in previous studies.

The result of VR intervention containing significant improvement in the self-control of children with ADHD seems to accord with the study of Witowska et al [42], which suggested that VR interventions can promote sustained focus and attention, which are essential components of self-control, by presenting engaging and visually stimulating tasks. The immediate feedback loop generated by the VR system can help children with ADHD understand the relationship between their behaviors and outcomes, facilitating self-monitoring and self-regulation. VR interventions can promote self-control by reinforcing desired behaviors and highlighting the impact of impulsive or impulsive actions.

This study found that VR-based intervention could improve the initiative of children with ADHD. This may be attributable to the sense of autonomy and self-determination that VR provides, allowing children to make choices and decisions within the digital environment [43]. The opportunity to experience success and skill development through VR-based feedback and reinforcement can also boost children’s confidence and willingness to initiate social interactions [39,44]. Additionally, interaction with digital characters or avatars may foster a sense of relatedness and social connectedness, encouraging children to practice and refine their social skills within the VR environment [45].

Contrary to expectations, no significant differences were found between VR training and traditional social skills training in terms of inhibition and cooperation. This differs from previous suggestions that digital classroom remediation could improve the inhibition of children with ADHD [18]. The absence of peer involvement in this study, as opposed to the successful peer interactions emphasized in prior research [46], maybe a factor contributing to the lack of differentiation between the 2 training approaches in enhancing cooperation. Further research is needed to directly compare the effects of VR-based interventions on self-control, initiative, inhibition, and cooperation across diverse populations, including children with ADHD, individuals with autism spectrum disorder, and neurotypical adults, to elucidate the specific mechanisms and contextual factors that influence the efficacy of VR-based approaches for different groups.

While the results of this study appear to be consistent with the previous studies on the positive effects of VR-based intervention on emotional control [47,48], it is important to note that the current sample of children with ADHD may respond differently to the VR intervention compared to other populations, such as individuals with autism spectrum disorder or neurotypical adults. Differences in cognitive profiles, developmental stages, and specific challenges faced by these groups could lead to variations in the effectiveness of the VR-based approach.

It was suggested that VR can evoke emotional experiences through a sense of “being there” in the digital environment [49]. VR alters individual experiences by motivating users to try new things and allowing them to alter habitual emotional responses [50]. The sample groups of previous studies were different from ours in that their participants were adults and older adults; thus, the analyses must be interpreted with caution. Further research is needed to directly compare the effects of VR-based interventions on self-control and related outcomes across diverse populations, including children with ADHD, individuals with autism spectrum disorder, and neurotypical adults. Such comparative studies would help elucidate the specific mechanisms and contextual factors that contribute to the efficacy of VR-based approaches for different groups.

An important consideration for this study is the cost-effectiveness of the VR-based social skills intervention compared to the traditional in-person approach. While a full cost-effectiveness analysis was beyond the scope of this study, we were able to gather some preliminary data on the relative costs of the 2 interventions. The VR-based intervention required 1 part-time research assistant at a rate of HK $70 (US $9) per hour to guide each participant through the digital scenarios. In contrast, the traditional in-person training involved 1 teacher with a special education background at a rate of HK $200 (US $25.74) per hour for each participant. This suggests the VR-based approach may be significantly more cost-effective, requiring less specialized personnel time per participant. Additionally, the VR equipment and software can be reused across multiple participants, whereas the in-person training requires the teacher’s time for each session. This further implies the VR intervention could have lower marginal costs as the number of participants increases. While these initial cost comparisons are promising, a more rigorous cost-effectiveness analysis would be needed to fully evaluate the economic feasibility of implementing VR-based social skills training on a larger scale. Factors such as the upfront investment in VR hardware, software licensing fees, and training for research assistants would also need to be considered. Future research should explore these cost implications in more depth to comprehensively assess the economic viability of VR-based social skills interventions compared to traditional approaches.

In summary, our findings suggest that creating VR environments with different social interaction scenarios for children with ADHD may have some practical implications. VR can be particularly beneficial in improving basic social skills, self-control, initiative, and emotional control, although it was less effective in enhancing inhibition and cooperation in children with ADHD. Thus, VR may be appropriate for providing training in social skills, self-control, initiative, and emotional control. Additionally, children with ADHD reported high levels of satisfaction and motivation during short-term VR-based interventions, suggesting that VR training could be a useful first phase in assisting these children in acquiring social skills in a community setting.

### Limitations

Although the effects of VR training are evident, potential limitations should be considered when interpreting the results. First, the small sample size recruited for this study may have limited the statistical power to determine differences between groups; nevertheless, this study provides important insights. Second, the design of this RCT had no follow-up period, and thus, the long-term effects after the intervention are unknown. Third, the trial was conducted with a highly specific type of population, and the results may not generalize to more complex ADHD populations. Fourth, a key limitation was that the satisfaction of the participants in the traditional social skills training group was not measured. Evaluating the participants’ satisfaction, engagement, and perceptions of the traditional training approach would have provided valuable comparative insights. Without this information, it is difficult to fully assess the relative benefits and drawbacks of VR training compared to the standard intervention from the participants’ perspectives. Future studies should consider incorporating participant satisfaction measures for all intervention groups to enable a more comprehensive evaluation of the different training methods. Fifth, although age differences across groups were found to be insignificant, the sample size within multiple age groups was limited, with some age groups having only 1 participant. This uneven distribution restricts our ability to conduct robust subgroup analyses to understand the differential effects of VR-based intervention versus traditional social skills training on social skills across various age groups. Given that social skills in children with ADHD are known to improve with age, this limitation highlights a significant knowledge gap in our study. Future research should aim to recruit a larger and more evenly distributed sample across different age groups to enable more detailed age-specific analyses. Finally, the sample size of this study may not provide sufficient statistical power for conducting subgroup analyses. This limitation means that our findings primarily reflect general comparisons between the intervention groups rather than nuanced insights into how different age groups respond to VR-based intervention versus traditional social skills training. Researchers should consider this limitation when interpreting our results and should design future studies with larger sample sizes to ensure adequate power for subgroup analyses.

### Conclusions

This RCT investigated the feasibility and effectiveness of using VR in improving the social skills of children aged 6-12 years with ADHD. The results showed that VR training was more effective than traditional social skills training in terms of enhancing basic social skills, self-control, initiative, and emotional control. VR and traditional social skills training can enhance basic social skills, self-control, initiative, and emotional control in children with ADHD. These encouraging findings require further confirmation with large-scale interventions to determine the sustainability of the effects. Continued research in this area will contribute to the development of evidence-based practices and interventions that can ultimately improve the social outcomes and quality of life for this vulnerable population.

## Appendix

Multimedia Appendix 1 CONSORT-eHEALTH checklist (V 1.6.1).

Multimedia Appendix 2 Description of the 3 scenarios in the virtual reality intervention.

Multimedia Appendix 3 Participants conducting the virtual reality–based intervention.

## Abbreviations

ADHD: attention-deficit/hyperactivity disorder
CONSORT: Consolidated Standards of Reporting Trials
RCT: randomized controlled trial
VR: virtual reality
