# Supplementary material for: Empowering Social Growth Through Virtual Reality–Based Intervention for Children With Attention-Deficit/Hyperactivity Disorder: 3-Arm Randomized Controlled Trial
Source: JMIR Serious Games. 2024 Oct 28;12:e58963. doi: 10.2196/58963 (PMC11555456; doi:10.2196/58963)
Supplement: Multimedia Appendix 2 [file games_v12i1e58963_app2.docx]

| Scenario | Description | Target improvement | Examples |
| --- | --- | --- | --- |
| MTR (Public Transportation) | - Participants take the MTR to the destinations required by the instructions. - Participants are required to abide by MTR etiquette and manners during the ride. - Various passengers and strangers ask the participants for help. - Multiple activities occur inside the compartment, training participants’ attention, initiative, and inhibition. - An RA acts as an avatar to guide the participants. - Multiple-choice questions: Select the correct choices and the colour of the option turns green. Select the wrong choice and the colour of the option turns red. | Learning the MTR manners and etiquette | The participant needs to select the right payment tool to enter the entry.  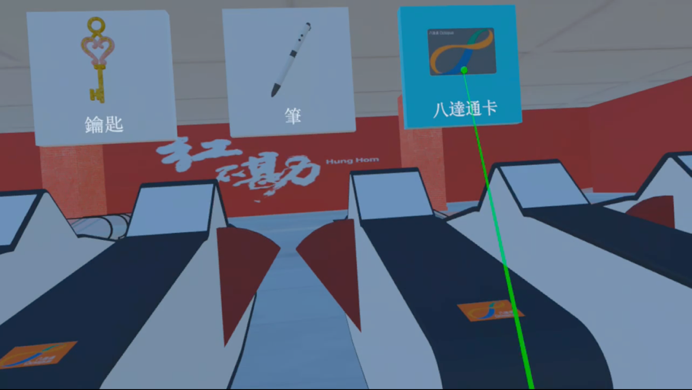  The participant as an Idle user generally keeps to the right.  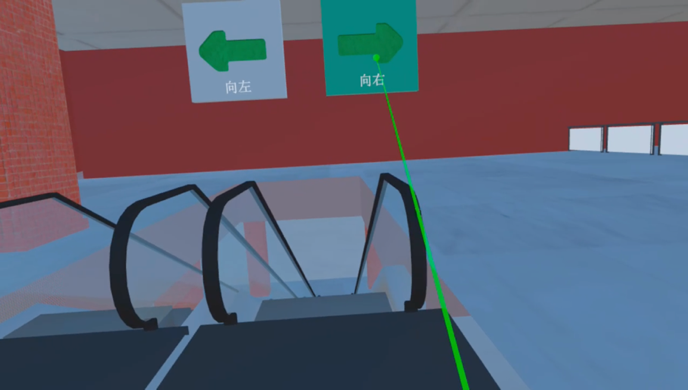  The participant needs to hold the handrail and stand firm when using the escalator.  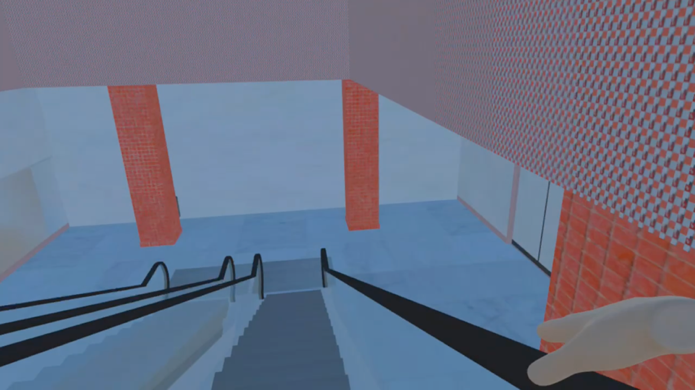  The participant has to wait in line.  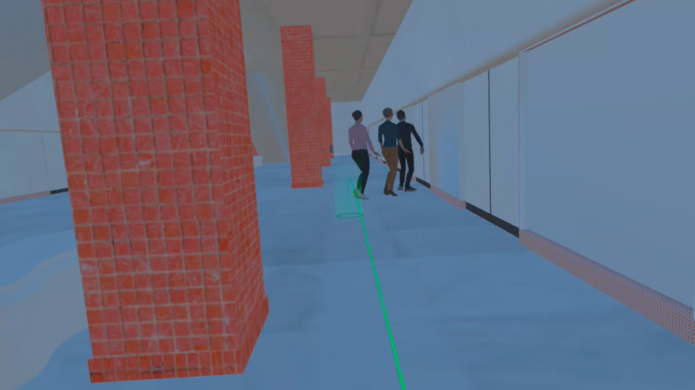  The participant has to let passengers exit first.  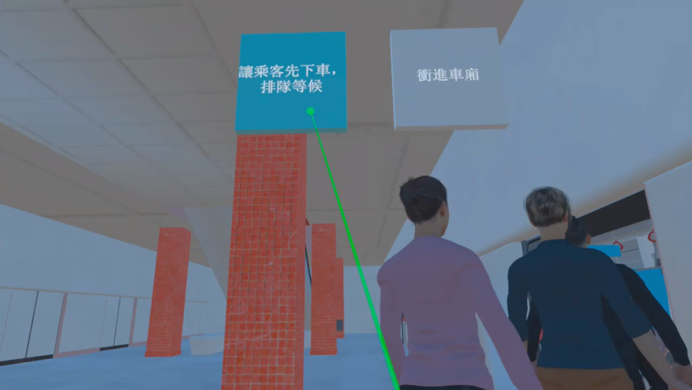  The participant needs to hold the handrail in the compartment.  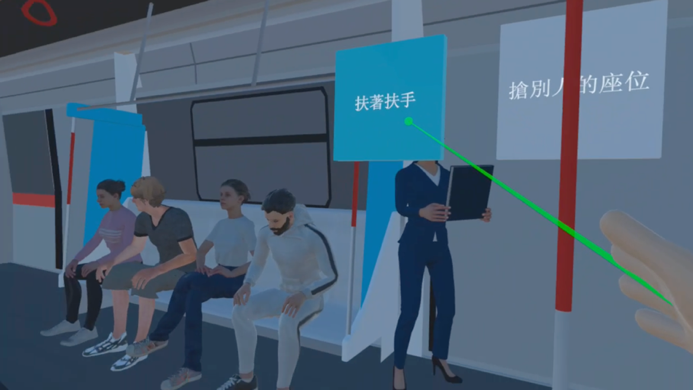 |
|  |  | Enhancing social interaction and initiative | The participant encountered different events, including:  Helping the elder adult to yield the seat.  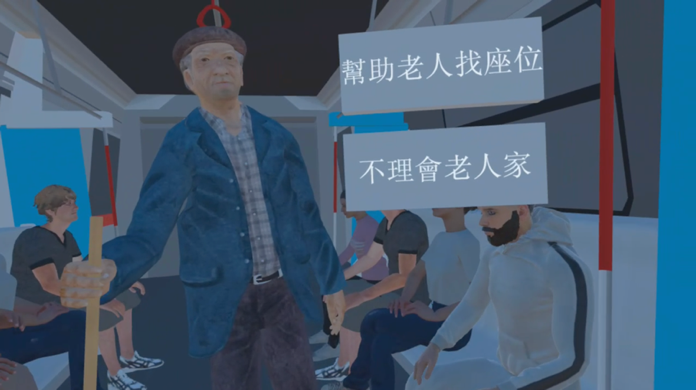  Helping the woman tidy up the scattered book and giving her back.  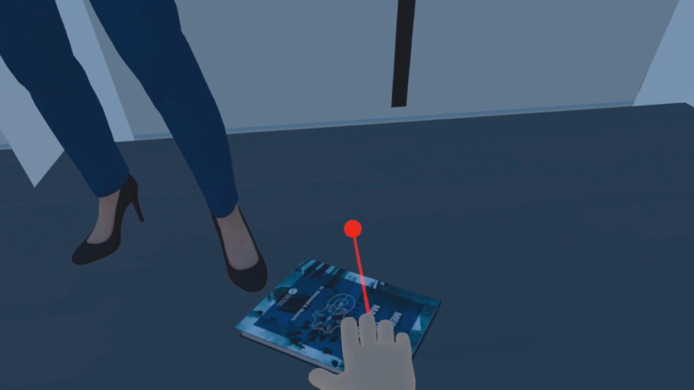  **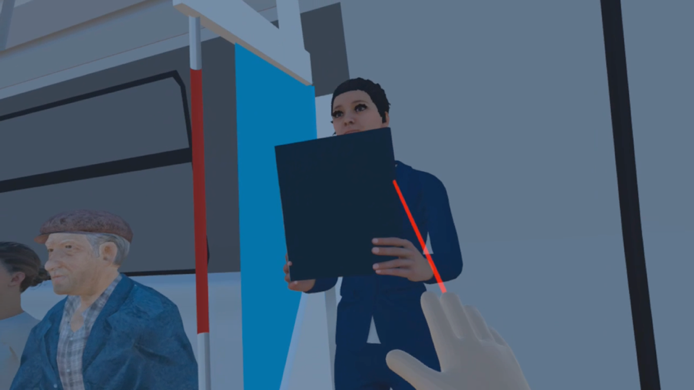**  Helping the pregnant woman to yield the seat.  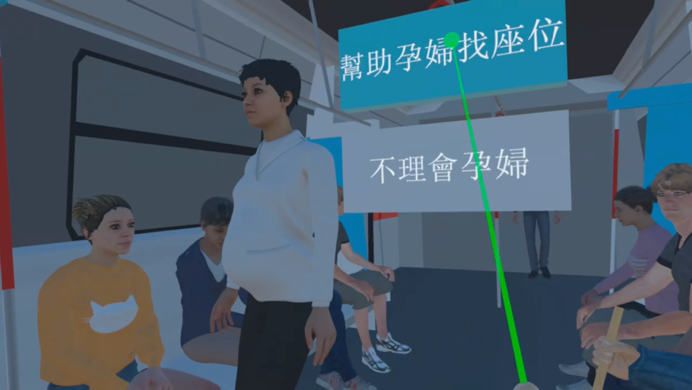  A passerby asked for directions.  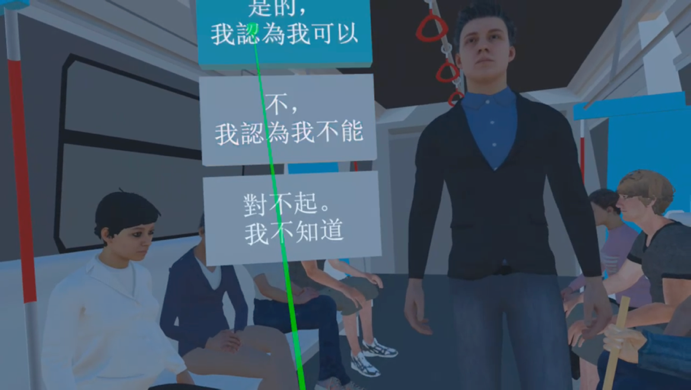  Purchasing a bottle of water in the 711-convenience store.  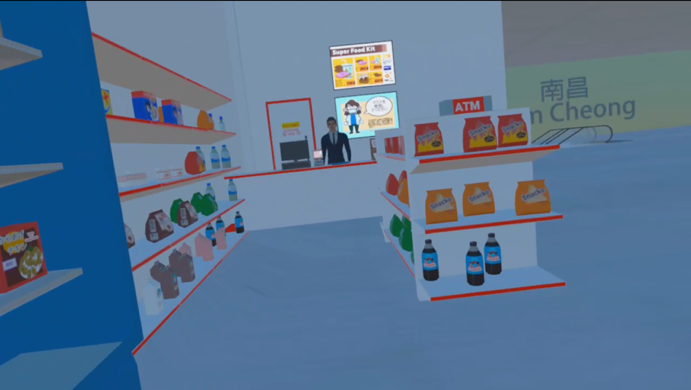 |
|  |  | Training attention and self-control | Children were chasing each other.  Participants need to choose to "play with them" or "queue".  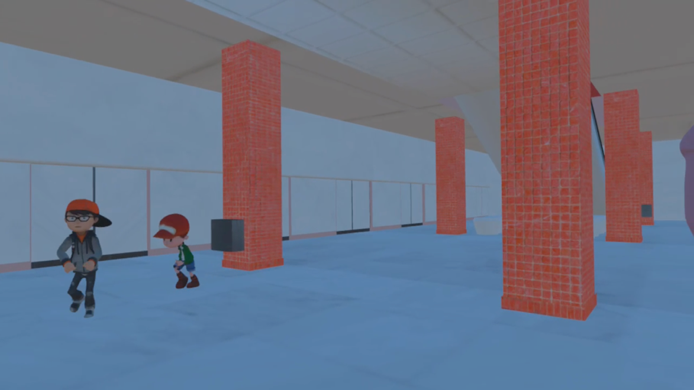 |
| Classroom and playground | - Participants follow the instructions of the teacher and complete each task accordingly. - The participants interact with his/ her classmates and teachers. - Participants encounter several incidents in the classroom and playground to train their attention, initiation, and inhibition. - An RA acts as an avatar to guide the participants. | Training the social interaction | The teacher taught the participant different greetings.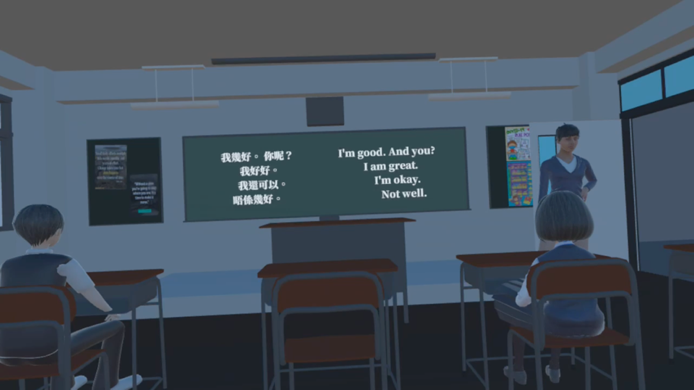  The teacher asked the participant to maintain order in thevclassroom.  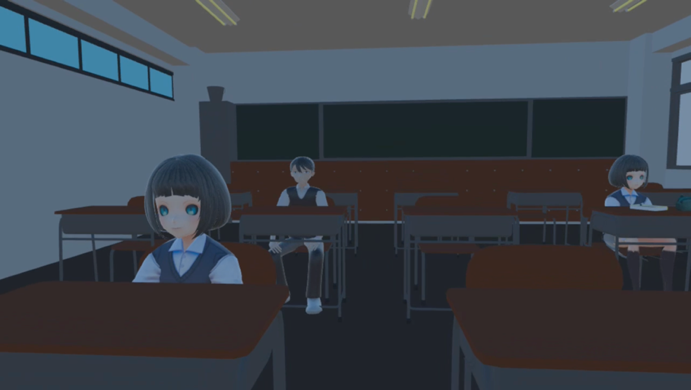  A classmate shared a drink with the participant.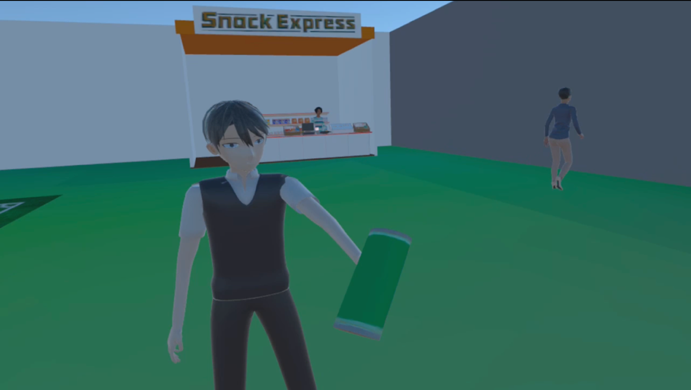  The school tuckshop sale asked the participant if he/ she needed anything.  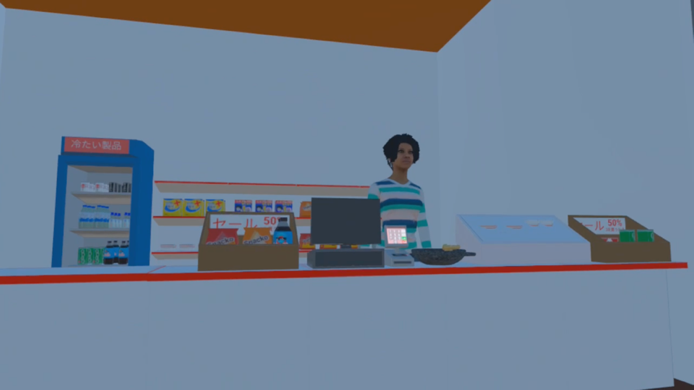  The classmate shared her fish balls with the participant.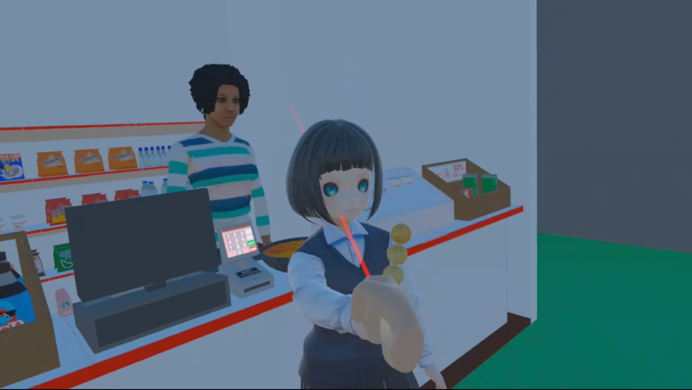 |
|  |  | Training attention and self-control | A bird singing by the window  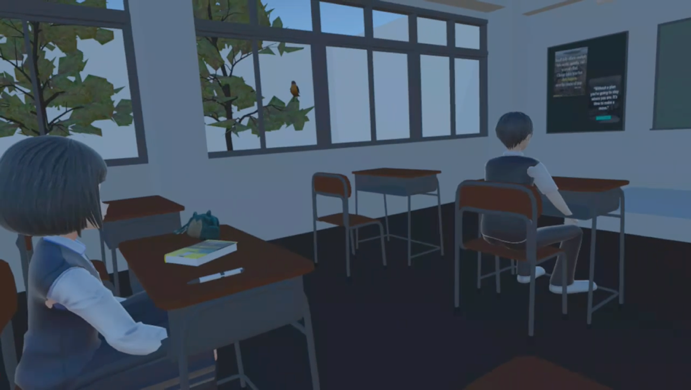  A classmate chatted with the participant during the lesson.  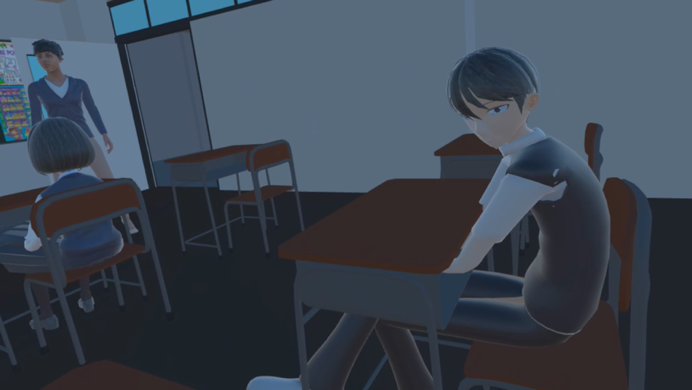  A man suddenly ran into the classroom and said hello.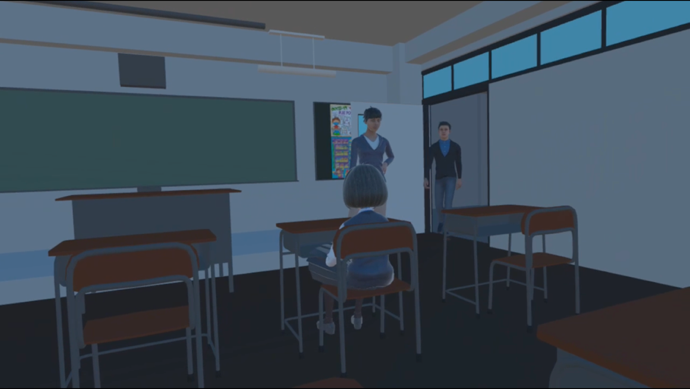 |
|  |  | Training the initiative | The classmate’s pencil next to me fell to the ground.  The participant helped the classmate pick the pencil up.  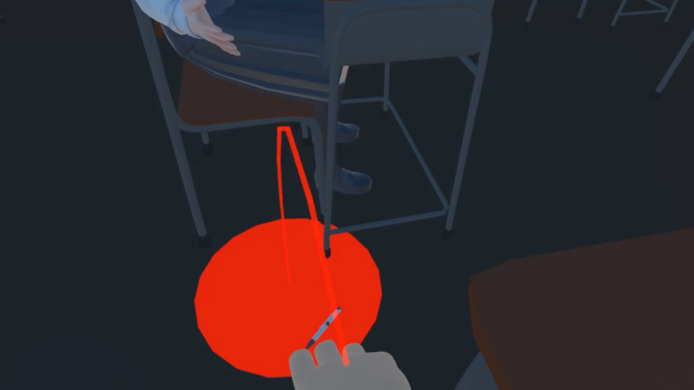  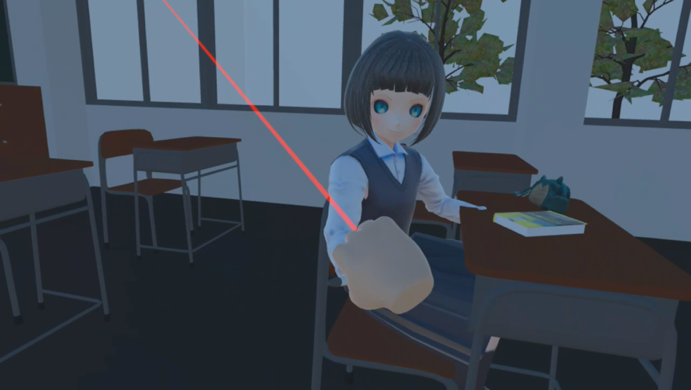 |
|  |  | Learning school manner | When the bell rang, the students lined up to follow the teacher to the playground.  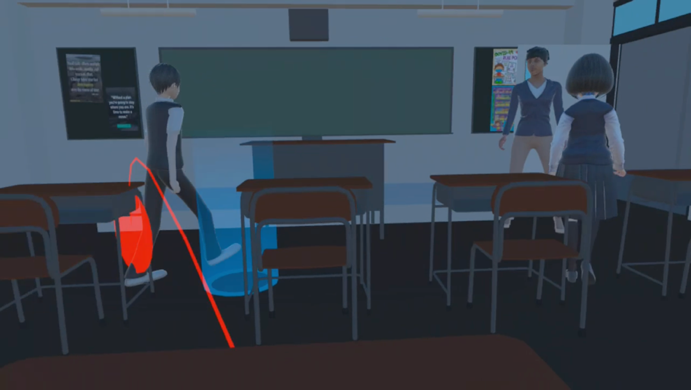  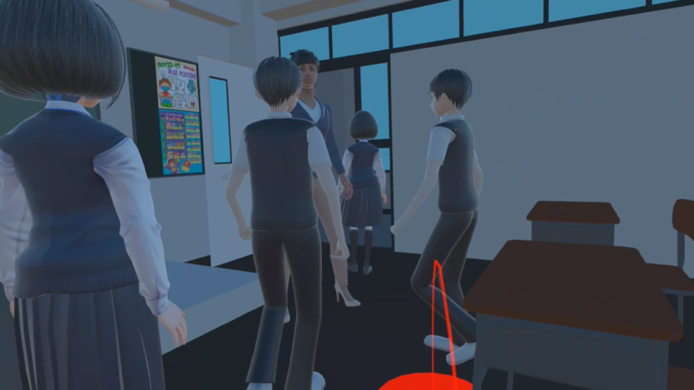  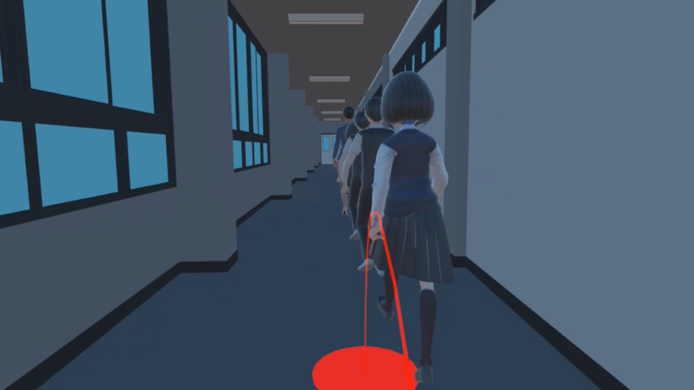  When the bell rang, the students lined up to follow the teacher to the classroom.  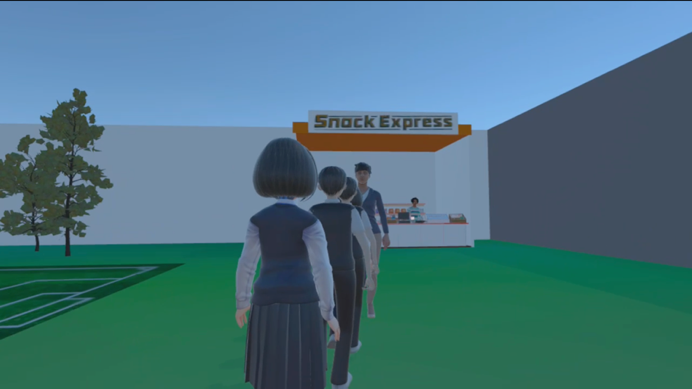  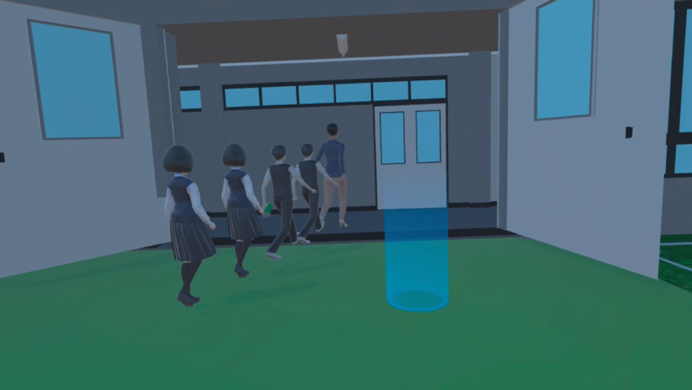 |
| Supermarket and Fast Food Shop | - Participants purchase some items at the market and buy takeaway food at the restaurant according to the instructions. - Participants interact with the salespeople and waiters. - The items are obtained and the items on the list are deleted. - An RA acts as an avatar to guide the participants. | Improving attention | The participant needs to purchase a list of items.  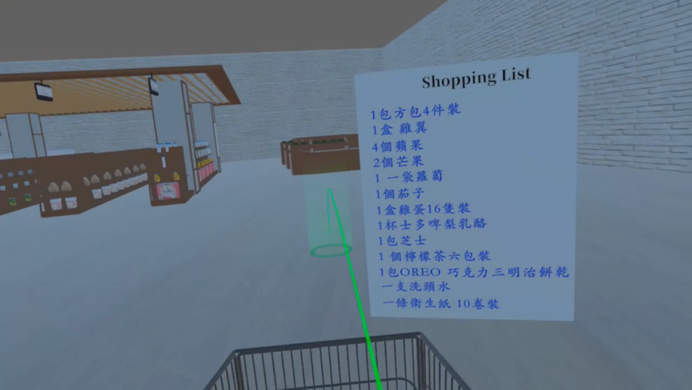  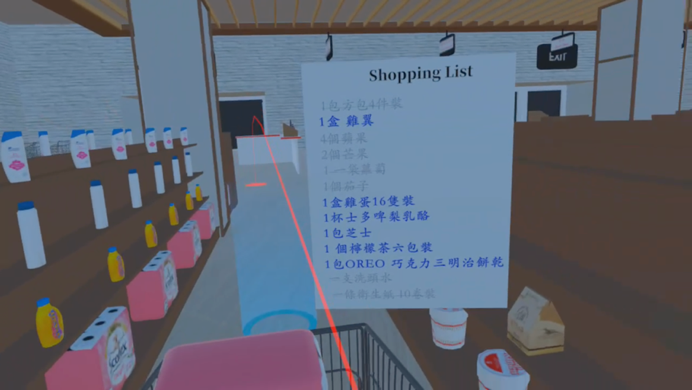 |
|  |  | Training the social interaction | The participant had some interaction with the salesperson during the payment process.  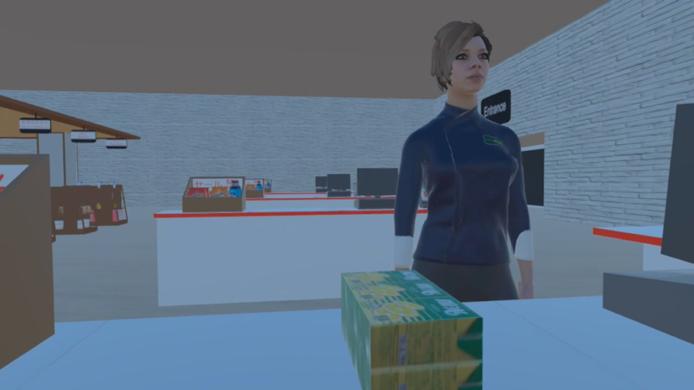  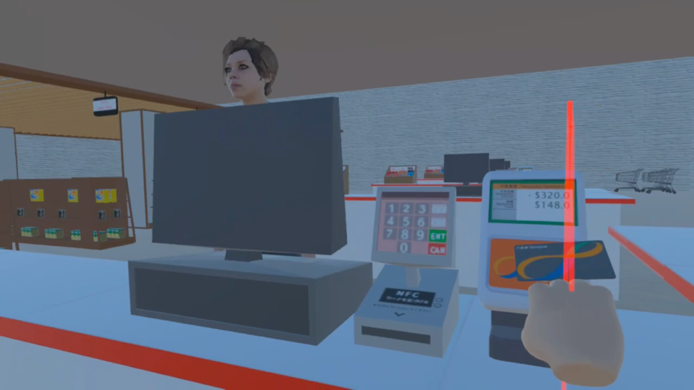  The participant had some interaction with the waitress when ordering the food.  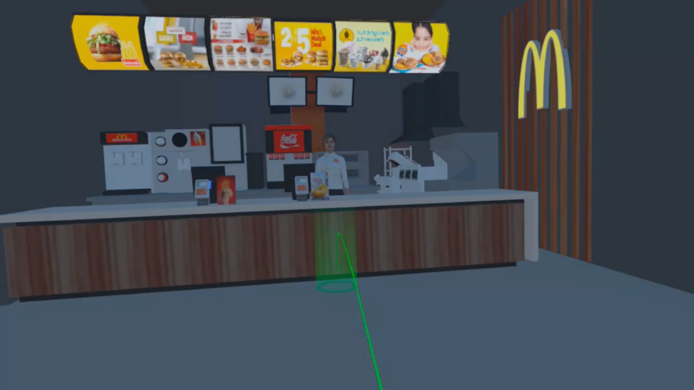  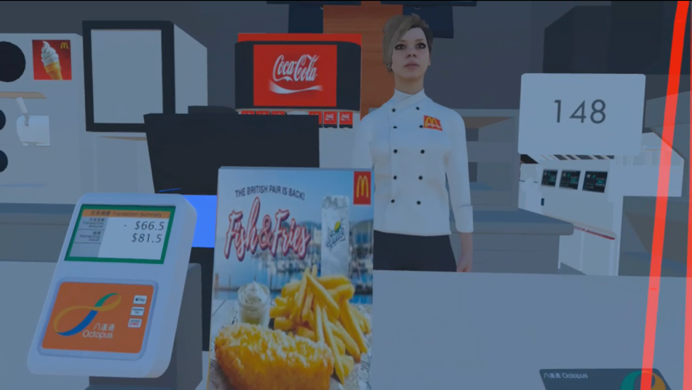 |
|  |  | Learning to cross the road safely | The participant needs to look at the traffic lights when crossing the road.  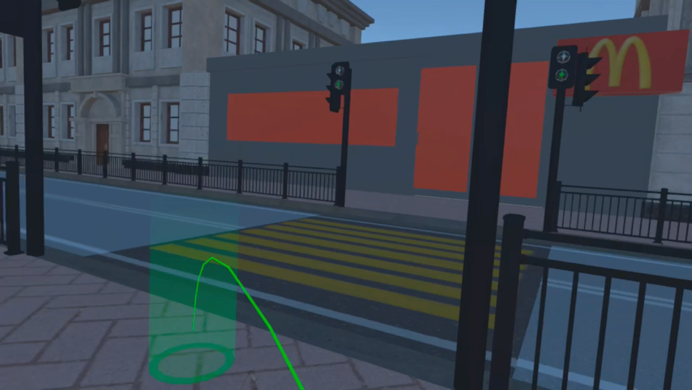 |
