# Supplementary figures and images for: Empowering Social Growth Through Virtual Reality–Based Intervention for Children With Attention-Deficit/Hyperactivity Disorder: 3-Arm Randomized Controlled Trial
Source: JMIR Serious Games. 2024 Oct 28;12:e58963. doi: 10.2196/58963 (PMC11555456; doi:10.2196/58963)

Multimedia Appendix 3. Participants conducting virtual reality-based intervention


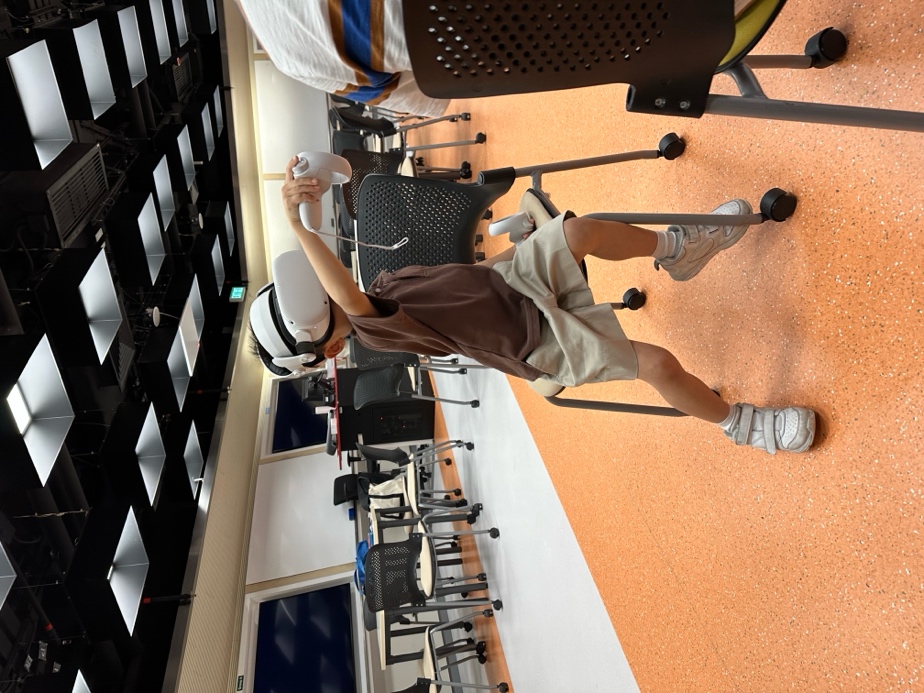

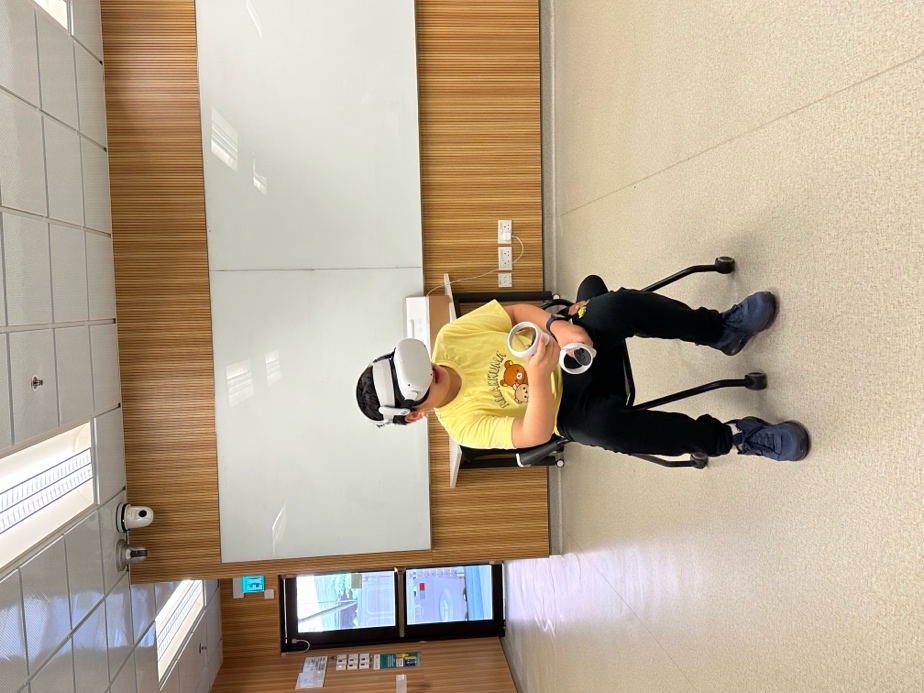

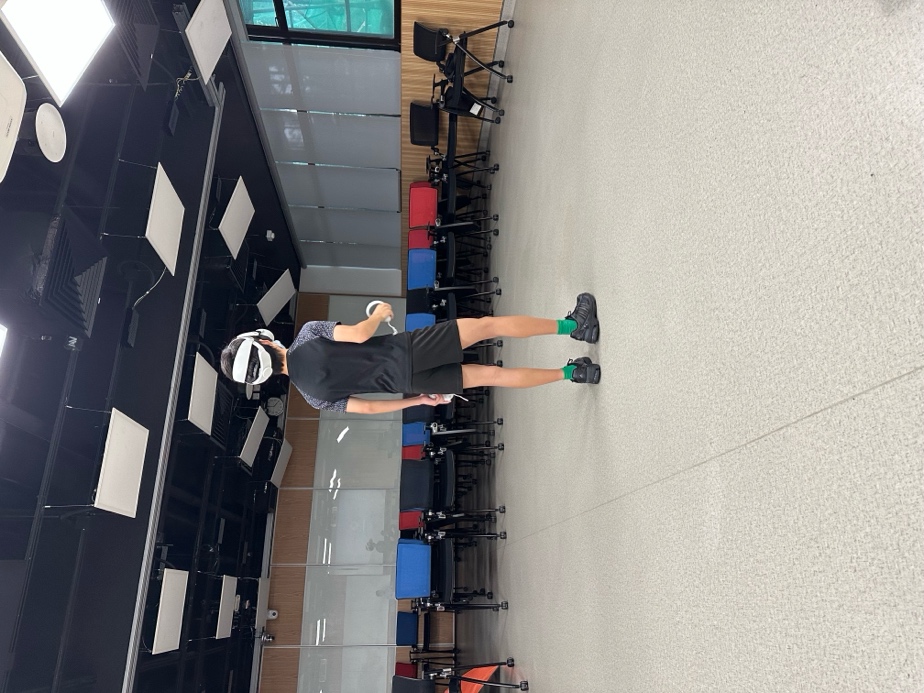

Supplement: Multimedia Appendix 3 [file games_v12i1e58963_app3.docx]
